# RNA sequencing reveals an additional Crz1-binding motif in promoters of its target genes in the human fungal pathogen *Candida albicans*

**DOI:** 10.1186/s12964-019-0473-9

**Published:** 2020-01-03

**Authors:** Huihui Xu, Tianshu Fang, Raha Parvizi Omran, Malcolm Whiteway, Linghuo Jiang

**Affiliations:** 1 Laboratory for Yeast Molecular and Cell Biology, Department of Food Science, School of Agricultural Engineering and Food Science, Shandong University of Technology, Zibo, 255000 China; 2 Department of Biology, Concordia University, Montreal, Quebec, H4B 1R6 Canada

**Keywords:** *Candida albicans*, Crz1, Calcium signaling, Transcription profiling, RNA sequencing, Promoter

## Abstract

**Background:**

The calcium/calcineurin signaling pathway is mediated by the transcription factors NFAT (nuclear factor of activated T cells) in mammals and Crz1 (calcineurin-responsive zinc finger 1) in yeasts and other lower eukaryotes. A previous microarray analysis identified a putative Crz1-binding motif in promoters of its target genes in *Candida albicans*, but it has not been experimentally demonstrated.

**Methods:**

An inactivation mutant for *CaCRZ1* was generated through CRISPR/Cas9 approach. Transcript profiling was carried out by RNA sequencing of the wild type and the inactivation mutant for *CaCRZ1* in response to 0.2 M CaCl_2_. Gene promoters were scanned by the online MEME (Multiple Em for Motif Elicitation) software. Gel electrophoretic mobility shift assay (EMSA) and chromatin immunoprecipitation (ChIP) analysis were used for in vitro and in vivo CaCrz1-binding experiments, respectively.

**Results:**

RNA sequencing reveals that expression of 219 genes is positively, and expression of 59 genes is negatively, controlled by CaCrz1 in response to calcium stress. These genes function in metabolism, cell cycling, protein fate, cellular transport, signal transduction, transcription, and cell wall biogenesis. Forty of these positively regulated 219 genes have previously been identified by DNA microarray analysis. Promoter analysis of these common 40 genes reveals a consensus motif [5′-GGAGGC(G/A)C(T/A)G-3′], which is different from the putative CaCrz1-binding motif [5′-G(C/T)GGT-3′] identified in the previous study, but similar to *Saccharomyces cerevisiae* ScCrz1-binding motif [5′-GNGGC(G/T)CA-3′]. EMSA and ChIP assays indicate that CaCrz1 binds in vitro and in vivo to both motifs in the promoter of its target gene *CaUTR2*. Promoter mutagenesis demonstrates that these two CaCrz1-binding motifs play additive roles in the regulation of *CaUTR2* expression. In addition, the *CaCRZ1* gene is positively regulated by CaCrz1. CaCrz1 can bind in vitro and in vivo to its own promoter, suggesting an autoregulatory mechanism for *CaCRZ1* expression.

**Conclusions:**

CaCrz1 differentially binds to promoters of its target genes to regulate their expression in response to calcium stress. CaCrz1 also regulates its own expression through the 5′-TGAGGGACTG-3′ site in its promoter.

Video abstract

## Plain English summary

Calcium ions regulate many cellular processes in both prokaryotes and eukaryotes, from bacteria to humans. Regulation of intracellular calcium homeostasis is highly conserved in eukaryotic cells. Gene expression in response to calcium stress is controlled by the calcium/calcineurin signalling through the transcription factors NFAT (the nuclear factor of activated T cells) in mammals and Crz1 (calcineurin-responsive zinc finger 1) in yeasts and other lower eukaryotes. Extracellular calcium stress causes an increase in cytosolic calcium, which leads to the binding of calcium ions to calmodulin that triggers activation of the protein phosphatase, calcineurin. Activated calcineurin dephosphorylates Crz1 in the cytosol, which leads to nuclear localization of Crz1 and its binding to promoters of its target genes to regulate their expression. *Candida albicans* is one of the most important human yeast pathogens. A previous microarray analysis identified a putative CaCrz1-binding motif in promoters of its target genes in *C. albicans*, but it has not been experimentally demonstrated. Using a new technology, RNA sequencing, we have identified 219 genes that are positively, and 59 genes that are negatively, controlled by CaCrz1 in response to calcium stress in this study. We have also revealed and demonstrated experimentally a novel consensus CaCrz1-binding motif [5′-GGAGGC(G/A)C(T/A)G-3′] in promoters of its target genes. In addition, we have discovered that CaCrz1 can bind to its own promoter, suggesting an autoregulatory mechanism for *CaCRZ1* expression. These findings would contribute to our further understanding of molecular mechanisms regulating calcium homeostasis.

## Backgound

Calcium ions regulate many cellular processes in both prokaryotes and eukaryotes, from bacteria to humans [1–5]. Intracellular calcium homeostasis is maintained by calcium transporters and sequestrators in the plasma and organelle membranes in eukaryotes. Regulation of calcium homeostasis is highly conserved in eukaryotic cells. Gene expression in response to calcium stress is controlled by the calcium/calcineurin signalling through the transcription factor Crz1 in fungi or the nuclear factor of activated T cells (NFAT) in mammals [6, 7]. In *Saccharomyces cerevisiae*, an increase in cytosolic calcium triggers the calmodulin/Ca^2+^ binding and activation of the protein phosphatase, calcineurin. Activated calcineurin dephosphorylates ScCrz1 in the cytosol, which leads to nuclear localization of ScCrz1 and its binding to promoters of its target genes, including the vacuolar calcium pump gene *ScPMC1*, the ER/Golgi calcium pump gene *ScPMR1* and the *ScRCH1* gene encoding the negative regulator of calcium uptake in the plasma membrane [8–10]. A genome-scale genetic screen has revealed additional genes that are involved in the regulation of calcium homeostasis in budding yeast [11].

*Candida albicans* remains as one of leading human fungal pathogens in immunocompromised patients [12–14]. Functional counterparts of calcium homeostasis and calcium/calcineurin signaling components have been characterized in *C. albicans* [15–21]. The calcium/calcineurin signaling functions in ion homeostasis, cell wall biogenesis, morphogenesis and drug resistance in *C. albicans* [22–24]. *C. albicans* cells lacking calcineurin show significantly reduced virulence in a murine model of systemic infection and fail to survive in the presence of membrane stress [25–27]. However, *C. albicans* cells lacking *CaCRZ1*, the major target of calcineurin, are partially virulent in the CAF4–2 strain background and even not virulent in the BWP17 background in the mouse model of systemic infection [28, 29]. Therefore, other targets are responsible for the calcineurin-mediated virulence in *C. albicans*. We have recently screened the GRACE (gene replacement and conditional expression) library of 2358 conditional mutants and identified a total of 21 genes whose conditional repression leads to the sensitivity of *C. albicans* cells to high levels of extracellular calcium [30–32]. In addition to 3 reported genes, *CRZ1*, *MIT1* and *RCH1* [16, 20, 28, 33], the rest newly-identified 18 calcium tolerance-related genes are involved in tricarboxylic acid cycle, cell wall integrity pathway, cytokinesis, pH homeostasis, magnesium transport, and DNA damage response.

Microarray analysis indicates that calcium-induced upregulation of 60 genes with a putative CaCrz1-binding motif [5′-G(C/T)GGT-3′] is dependent on both calcineurin and CaCrz1 in *C. albicans* [28]. Both microarray and RNA sequencing are used to measure genome-wide transcriptomic changes in different organisms, and they complement to each other in transcriptome profiling [34–36]. However, RNA sequencing approach is much more sensitive than the microarray, with the dynamic range of the former reaching at least 8000-fold in comparison to the latter only at around 60-fold in expression levels of genes detected [37].Therefore, we have examined the regulatory function of CaCrz1 in gene expression with the RNA sequencing technology in this study. We show that expression of 219 genes is positively controlled, and expression of 59 genes is negatively controlled, by CaCrz1 in the SN148 background in response to calcium stress. Furthermore, we have revealed an additional CaCrz1-binding motif in promoters of its target genes and demonstrated that CaCrz1 binds to both motifs in the promoter of its target gene *CaUTR2*.

## Methods

### Strains and media

*C. albicans* strains and plasmids used in this study were described in Table 1. Primers used in this study were listed in Additional file 1: Table S1. Strains were grown and maintained at 30 °C in YPD medium or SD medium (0.67% yeast nitrogen base without amino acids, 2% glucose, and auxotrophic amino acids as needed). Chemicals were obtained from Sigma (USA) and Sangon Biotech (Shanghai, China).

**Table 1:** Strains and plasmids used in this study

Name	Genotype or Description	Source
Strain		
SN148	Mat*a/α arg4/arg4 leu2/leu2 his1/his1 ura3*::*imm434/ura3*::*imm434*	[38]
HHCA184	SN148 *crz1/crz1 ENO1/eno1:: natMX4*	This study
HHCA185	SN148 *crz1/crz1 ENO1/eno1:: natMX4*	This study
HHCA187	SN148 *crz1/crz1 ENO1/eno1:: natMX4*	This study
HHCA1	SN148 *RPS1/rps1::*CIp10	This study
HHCA2	HHCA184 *RPS1/rps1::*CIp10	This study
HHCA3	HHCA184 *RPS1/rps1::*CIp10-CaCRZ1	This study
Plasmid		
pV1093	*Amp*^*R*^ *Nat*^*R*^	[39]
pV1093-sgCRZ1	*Amp*^*R*^ *Nat*^*R*^	This study
CIp10	*Amp*^*R*^ *URA*	[38]
CIp10-sgCRZ1	*Amp*^*R*^ *URA*	This study

### Construction of CRISPR mutant for *CaCRZ1*

*C. albicans* strain SN148 was used as the parent strain to construct the CRISPR inactivation mutant for *CaCRZ1* through the CRISPR [Clustered Regularly Interspaced Short Palindromic Repeat)/Cas9] approach (Additional file 1: Figure S1). We designed SgRNA primers CRZ1-sgF and CRZ1-sgR near the start codon of *CaCRZ1* using the online software Benchling (https://benchling.com/academic) as well as the repair DNA primers CRZ1-RFand CRZ1-RR containing 40-bp homologous regions flanking the SgRNA sequence (Additional file 1 : Figure. S1). Primers CRZ1-sgF and CRZ1-sgR were annealed, cut with *Bsm*BI and cloned into the *Bsm*BI site of pV1093 (Additional file 1: Figure S1A-S1B), which generated the recombinant plasmid pV1093-SgRNA. SgRNA sequence in pV1093-SgRNA was confirmed by DNA sequencing. Primers CRZ1-RF and CRZ1-RR were annealed for PCR amplification of the repair DNA fragment of about 100 bp. The repair DNA and the recombinant plasmid pV1093-SgRNA linearized by both *Sac*I and *Kpn*I were used together to transform cells of *C. albicans* strain SN148 (Additional file 1: Figure S1C). Potential correct CRISPR mutants for *CaCRZ1* were detected with diagnostical *Pst*I-digestion of 1-kb PCR products, containing the SgRNA region, amplified with primers CRZ1-CF and CRZ1-CR from genomic DNA samples of transformants (Additional file 1: Figure S1D-S1E). Mutated sites in *CaCRZ1* alleles in those potential correct CRISPR mutants were further confirmed by DNA sequencing.

### DNA manipulation

To clone the full-length gene *CaCRZ1* into the integration vector CIp10 [40], a DNA fragment containing the 758-bp promoter, the 2196-bp open reading frame (ORF) and the 336-bp terminator region of *CaCRZ1* was amplified with primers CRZ1-clonF and CRZ1-clonR, and cloned between *Kpn*I and *Xho*I sites in the CIp10, which yielded CIp10-CaCRZ1. To do complementation experiment, the wild type and the *crz1/crz1* mutant strains were integrated with the *Stu*I-linearized plasmids CIp10 or CIp10-CaCRZ1, respectively, as described [41].

To express the His6-tagged CaCrz1 expression plasmid in bacterial cells, we first optimized the codon usage by mutating all five CTG codons in *CaCRZ1* to TCT codon (L22S), AGC codon (L24S), TCC codons (L601S, L649S and L686S) (Additional file 1: Fig. S2). The codon-optimized open reading frame (ORF) of CaCRZ1 was artificially synthesized and cloned into the vector pET28a(+), which yielded pET28a(+)-CRZ1 that expressing the codon-optimized and N-terminally Hisx6 tagged full-length CaCrz1 (His6-CaCrz1) protein. The pET28a(+)-CRZ1 was introduced and expressed in BL21(DE3) bacterial cells as described [42–44].

To construct a *lac*Z reporter plasmid, the bacterial *lac*Z gene was first amplified with a pair of primers lacZ_ORF_F(XhoI) and lacZ_ORF_R(KpnI) from the plasmid pGP8 [15, 28], and cloned into the *Kpn*I and *Xho*I sites of CIp10 to yield CIp10-lacZ. The terminator of *CaACT1* was amplified from the SN148 genomic DNA with two primers ACT1_T_F(KpnI) and ACT1_T_R(KpnI), and cloned into the *Kpn*I site of CIp10-lacZ to yield CIp10-lacZ-T_ACT1_. The *CaUTR2* promoter was amplified from the SN148 genomic DNA with a pair of primers UTR2_P_F(XhoI) and UTR2_P_R(XhoI) and cloned into the *Xho*I site of CIp10-lacZ-T_ACT1_ to yield CIp10-UTR2-lacZ.

To mutate the putative CaCrz1-binding motif identified in our study, the underlined sequence in the 5′-TCT(− 343) CAACGCCTCA(− 333)AAA-3′ region of *CaUTR2* promoter was mutated to be 5′-TCT(− 343)TCTAGA(− 333)AAA-3′ (we designated this mutation as UTR2(HΔ)), which contains a *Xba*I site. This was accomplished by a fusion PCR strategy. We first amplified the upstream (A) and downstream (B) fragments of the *CaUTR2* promoter with two pairs of primers UTR2_exF/ UTR2_(HΔ)_R and UTR2_inR/ UTR2_(HΔ)_F, respectively. These two fragments (A and B) were then fused by PCR with the two primers UTR2_P_F(XhoI) and UTR2_P_R(XhoI), and cloned into the *Xho*I site of CIp10-lacZ-T_ACT1_ to yield CIp10-UTR2(HΔ)-lacZ. Similarly, to mutate the putative CaCrz1-binding motif identified in the previous study [28], the underlined sequence in the (5′-TTGT(− 377)GGGCTT(− 371)TGA-3′ region of *CaUTR2* promoter was mutated to be (5′-TTGT(− 377)TCTAGAT(− 371)TGA-3′ (we designated this mutation as UTR2(MΔ)), which contains a *Xba*I site. The upstream (C) and downstream (D) fragments of the *CaUTR2* promoter were first PCR amplified with two pairs of primers UTR2_exF/ UTR2_(MΔ)_R and UTR2_inR/UTR2_(MΔ) _F, respectively. These two fragments (C and D) were then fused by PCR with the two primers UTR2_P_F(XhoI) and UTR2_P_R(XhoI), and cloned into the *Xho*I site of CIp10-lacZ-T_ACT1_ to yield CIp10-UTR2(MΔ)-lacZ. To create the CIp10-UTR2(HMΔ)-lacZ with mutations for both UTR2(HΔ) and UTR2(MΔ) in the *CaUTR2* promoter, the two DNA fragments (A and D) were fused by PCR with primers UTR2_P_F(XhoI)/ UTR2_P_R(XhoI), and cloned into the XhoI site of CIp10-lacZ-T_ACT1_. Inserts in all recombinant plasmids were confirmed by DNA sequencing.

### RNA sequencing and data analysis

To identify genes regulated by CaCrz1, the wild type SN148 and its isogenic CRISPR mutant for *CaCRZ1* were grown to log-phase at 30 °C before they were treated with 0.2 M CaCl_2_ for 2 h. Total RNA samples were extracted Qiagen RNeasy minikit protocol, and RNA integrity was evaluated using an Agilent 2100 Bioanalyzer (Agilent Technologies, USA) as described [45]. RNA-seq libraries were constructed using Illumina’s miSEQ RNA Sample Preparation Kit (Illumina Inc., USA). RNA sequencing, data analysis and sequence assembly were performed by the Quebec Genome Innovation Center at McGill University (Montreal, Canada) [31, 38]. Preparation of the paired-end libraries and sequencing were performed following standard Illumina methods and protocols. The mRNA-seq library was sequenced using an Illumina miSEQ sequencing platform. Clean reads from RNA-Seq data were assembled into full-length transcriptome with the reference genome (http://www.candidagenome.org/). Functional categories of genes were carried out by the Munich Information Center for Protein Sequences (MIPS) analysis.

### Galactosidase activity assay

To measure the *UTR2* promoter-driven β-galactosidase activity in the wild type and the *crz1/crz1* mutant, we integrated the *Stu*I-linearized plasmids containing the *lac*Z reporters for *CaUTR2* promoter into the *RPS1* locus of these strains as described [16, 28]. The β-galactosidase activity was determined using the substrate ONPG as described [46, 47]. Data are mean ± SD from six independent experiments. Significant differences were analysed by GraphPad Prism version 4.00. *P* values of < 0.05 were considered to be significant.

## Results

### Construction of the CRISPR mutant for *CaCRZ1*

**Fig. 1:**
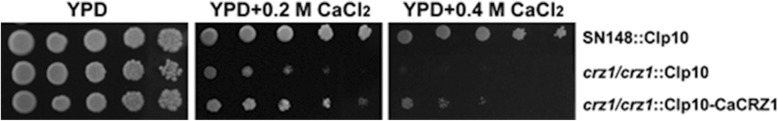
**Phenotypes of CRISPR mutant for** ***CaCRZ1*.** Cells of the wild-type SN148, the CRISPR mutant and the complemented strain were grown at 30 °C in liquid YPD overnight, serially diluted by 10 times and spotted on YPD plates with or without supplemented reagents as indicated, respectively. Plates were incubated for 2–5 days at 30 °C. CsA, cyclosporine A

**Fig. 2:**
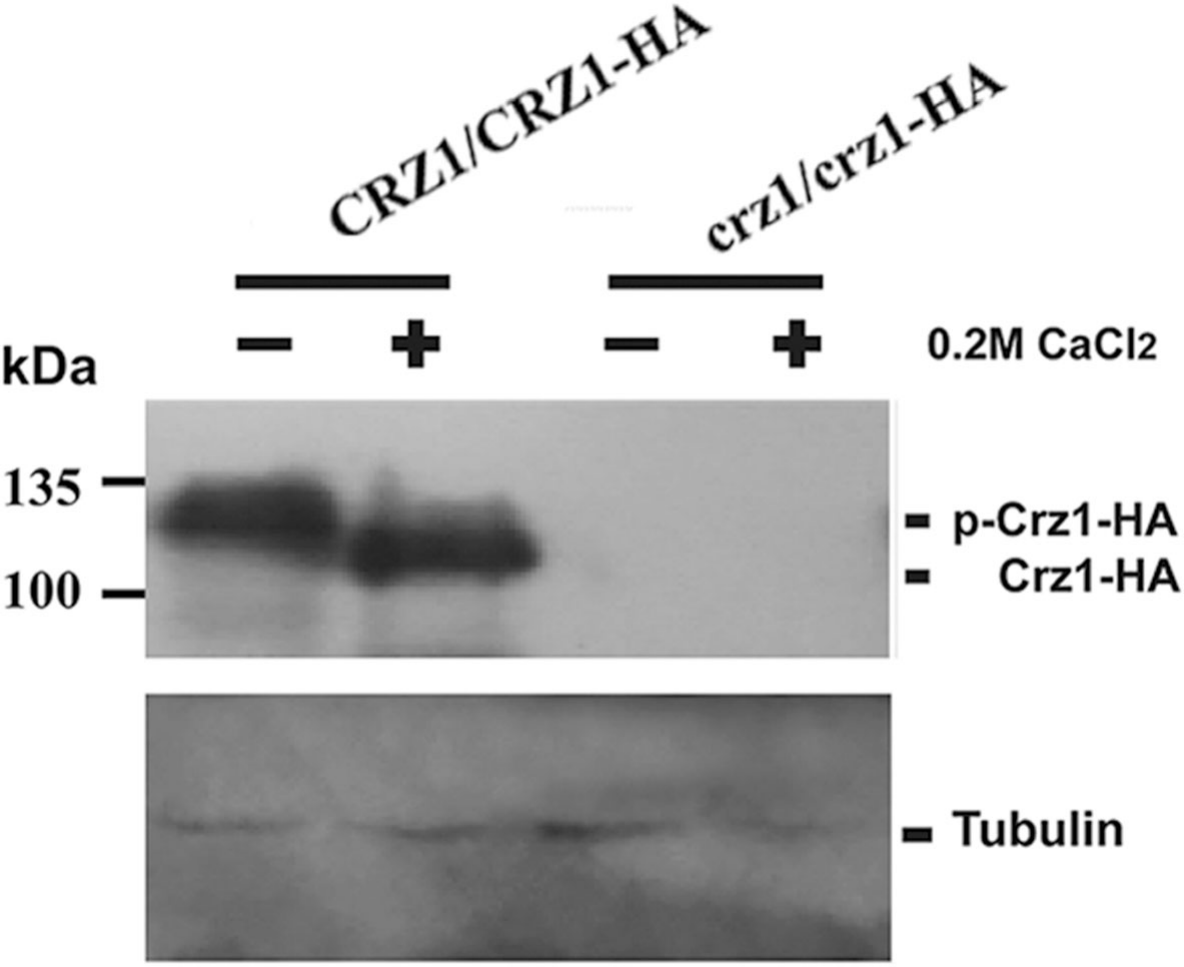
**Expression of the C-terminally HA-tagged CaCrz1 protein in** ***C. albicans*** **cells.** The wild type strain SN148 (*CRZ1/CRZ1*) and the CRISPR mutant for *CaCRZ1* (*crz1/crz1*) carrying their chromosomally C-terminally HA-tagged wild-type and mutated *CaCRZ1* alleles, respectively, were grown to log-phase in YPD medium at 30 °C before their cells were collected for total protein extraction. Expression of CaCrz1-HA proteins was detected by Western blot analysis with anti-HA monoclonal antibody. Expression of tubulin was detected using anti-tubulin antibody, which served as an internal expression control

To further study the regulatory functions of CaCrz1 in gene expression, we constructed three independent CRISPR mutants for *CaCRZ1* in the SN148 genetic background (Additional file 1: Figure S1A-S1E). These mutants were sensitive to 0.4 M CaCl_2_, and their calcium sensitivity was suppressed by the specific inhibitor of calcineurin, cyclosporine A. In addition, they were sensitive to 0.05% SDS, but not to antifungal drugs including clotrimazole, ketoconazole, fluconazole and terbinafine (Additional file 1: Figure S1F). These results agree with previous reports [21, 28, 29]. We chose one of these CRISPR mutants (HHCA184) for our RNA sequencing, and its calcium-sensitive phenotype could be partially reversed by the introduction of the *CaCRZ1* gene back to its genome (Fig. 1). To examine if the two mutated *CaCRZ1* alleles in the CRISPR mutant (HHCA184) were still able to express the CaCrz1 proteins in *C. albicans* cells, we chromosomally integrated the HA tag at the C-terminus of CaCrz1 in both the mutant and the wild type strain SN148. Through western blot analysis, we failed to detect the expression of CaCrz1-HA in the mutant, although we detected two forms of CaCrz1-HA proteins in the wild type, which might correspond to the phosphorylated form and dephosphorylated form of CaCrz1 (Fig. 2). Taken together, our data demonstrate that we have successfully constructed the CRISPR mutant for *CaCRZ1*.

### Transcriptomic profiling of cells lacking *CaCRZ1*

Next, we carried out transcript profiling for the wild type and the *crz1/crz1* mutant, growing in log phase in YPD medium at 30 °C in the absence or presence of 0.2 M CaCl_2_. Transcripts for two alleles of 6211 genes at various expression levels were detected in these two strains (SuppInfo 1; GEO Accession number: GSE123122). As compared to the wild type cells without 0.2 M CaCl_2_ treatment, there are 828 genes upregulated in the wild type cells with 0.2 M CaCl2 treatment, among which 219 genes are positively regulated, and 59 genes are negatively regulated, by CaCrz1 (SuppInfo 2; SuppInfo 3). These genes positively regulated by CaCrz1 play roles in metabolism (13), cellular transport (23), transcription (7), signal transduction (3), protein fate (17), cell rescue (9), cell cycle (6), cell fate/development/cell type differentiation (14) and cell wall biogenesis (34), with almost half of them (93) being of unknown functions (Table 2). In contrast, these genes negatively regulated by CaCrz1 function in metabolism [20], cellular transport [5], transcription [11] and cell wall biogenesis [3], with one third of them [20] being of unknown functions (Table 3). The *CaCRZ1* gene itself is positively regulated by CaCrz1, which is identified in both the previous microarray study and our current study (Table 2).

**Table 2:** Functional category of 219 genes positively regulated by CaCrz1 in response to 0.2 M CaCl2

Systemic name	Standard name	Systemic name	Standard name	Systemic name	Standard name	Systemic name	Standard name	Systemic name	Standard name
Metabolism (13)									
C1_04010C		C2_03640W	*UGA11*	C5_00220W	*ROT2*	C1_14060W		C1_11240C	*CHO1*
C2_01630W		C2_09150W	*MIT1*	CR_00620C	*ARG1*	C1_08330C	*ADH2*	C7_02500C	*DPP3*
C3_05810C	*SKN1*			C1_01620C					
Cellular transport (23)									
CR_03450W	*HXT5*	C5_04440C	*SFC1*	C3_03060W		C3_05270C	*HGT5*	C7_02910W	*ENA21*
C1_09220W		C2_09770C	*INP51*	C1_04630C				C7_00100W	*FRP2*
CR_05310W		C4_03110W		C3_07230W		C1_01100W	*CCH1*	C1_09400C	*FTH1*
C2_03800C		CR_09170C	*SSU1*	C2_06470W	*RTA2*	C2_07730W	*YVC1*	C1_06610C	*HAK1*
		CR_07100W	*FLC2*	CR_09680C	*RTA4*				
Transcription (7)									
CR_03890W	*WOR3*	C7_00970C	*YOX1*	C3_05780C	*CRZ1*	C1_05340C	*ZCF2*	C7_04230W	*NRG1*
CR_02300C		C4_04210C	*SOH1*						
Signal transduction (3)									
C5_02290W	*PDE1*	C4_06480C	*CEK1*	C7_00360W	*DFI1*				
Protein fate (folding, modification, destination) (17)									
C1_13220C	*AKR1*	C5_01440C		C2_00930C	*VPS24*	C5_01210W	*VPS1*	C4_03890W	*PTP2*
CR_00290W		C5_05060C		C4_04660C				C4_05810W	
C6_03500C	*SAP4*	C2_01670C	*STT3*	C4_00070C		C7_03250C	*PDI1*	CR_00260W	*KIN2*
C1_08170C	*BUL1*	C2_08790W	*JEM1*						
Cell rescue (9)									
CR_06040W		CR_01730W	*IFU5*	C2_02060C	*FMO1*	C2_00680C	*SOD5*	C1_02700C	
C2_09220W	*DDR48*	C3_00480C	*DOT5*	C2_05660W	*PNG2*	CR_05390W	*PST3*		
Cell Cycle (6)									
C1_09870W	*HCM1*	C6_03260W		C1_05170C	*CUE5*	C1_08570C	*PCL2*	C3_03850C	*SOL1*
C5_01680C	*CCN1*								
Cell fate/development/cell type differentiation (14)									
C4_03510C	*HWP2*	C7_00120W		C1_00850W	*IHD2*	C2_03040W	*PLC2*	C3_05190C	*MCA1*
C2_07930C	*VRP1*	C1_07770W	*FGR6–3*	C4_00600C	*MUC1*	C2_05260W	*BUD14*		
C6_00940C		C2_00080C	*FAV3*	C4_01010C	*DAG7*	C1_01440C	*POX18*		
Cell wall biosynthesis (34)									
C5_02630C	*MNN1*	CR_00740C	*BMT3*	C3_01730C	*UTR2*	CR_04440C	*RBR1*	C3_02140C	
C4_06540W	*MNN4*	C2_01560W	*BMT5*	CR_10480W	*PGA1*	C5_02460C	*ECM331*		
C1_04900W	*MNN15*	C3_03450C	*BMT7*	C1_09080C	*PGA6*	CR_03790C	*KRE1*	C4_02720C	
C2_01300C	*MNN24*	CR_00180C	*CHT1*	CR_08510W	*PGA13*	C6_01690W	*ACF2*		
C2_03690C	*MNN42*	C2_02010C	*CHT4*	CR_02280W	*PGA23*	C5_04110W	*SCW11*	C2_05040C	
C4_06990W	*MNN46*	CR_09020C	*CHS2*	CR_04900C	*PGA39*	C1_00220W	*PHR2*	C4_05100C	*MYO5*
C3_01830C	*MNT2*	C4_02900C	*CRH11*	C2_00100C	*PGA52*	C1_04000C	*KTR4*		
Unknown (93)									
CR_00380W		C3_07360W	*DLD2*	CR_10570C	*YHB4*	C3_04100W		C4_03590C	*OSH3*
C4_00410W		C3_07470W		C1_11970C		C2_09050C		C5_04330W	
C2_08620W		C4_06470W		C1_12060C		C2_10150W		C5_04470C	
C1_03870C		C5_03970W		C1_13240W		C2_10160W		C5_04480C	
CR_07160C		C2_08960C		C1_13590W		C2_10720C		C5_04540C	
C1_08610C		C1_00760W		C1_13810W		C3_02710W		C6_01250W	
C3_01550C	*TOS1*	C1_01510W		C2_00110W		C3_04190W		C6_02210W	
C5_04960W		C1_02370C		C2_00130W		C3_06670C		C6_04420W	
C7_01700W		C1_04440W		C2_00750W		C3_06680C		C7_00310C	
C1_03150C		C1_04470C		C2_00920W		C4_04190C		C7_00350C	
C1_09800C	*TVP18*	C1_05450W		C2_00940W		C4_04200C		C7_01390W	
		C1_05920W		C2_02220C		C4_05000W		C7_02370W	
CR_00420W		C1_07990C		C2_02900W		C4_05250W		C7_03310W	
		C1_08830C		C2_02910W		C4_05800C		CR_01020C	
CR_08470W		C1_10060C		C2_03570C		C4_07260W		CR_02880W	
C4_00860C		C1_10580C		C2_04750W		C5_00410W		CR_03780C	
C3_02570W		C1_11260C		C2_05120C		C5_03430W		CR_06550C	
C4_03870C		C1_11270W		C2_06630C		C5_04030W		CR_08990C	
CR_05460W		C4_00980C	*MRV1*	C2_08910C					

^**#**^Underlined genes are shared CaCrz1-regulated genes that has been identified by DNA microarray in a previous study [28]. Shadowed genes are sequence homologues for 9 *S. cerevisiae* genes positively regulated by ScCrz1 in response to 0.2 M CaCl2 reported in a previous study [48].-

**Table 3:** Functional category of 59 genes negatively regulated by CaCrz1 in response to 0.2 M CaCl2

Systemic name	Standard name	Systemic name	Standard name	Systemic name	Standard name	Systemic name	Standard name	Systemic name	Standard name
Metabolism (20)									
C6_00760W		C1_13870W	*MET3*	C4_00490W		C1_03820W	*PDR16*	CR_05340C	*IFE2*
C6_00620W	*FCA1*	C7_00490C		C5_05150C		C4_06950W			
C7_01600W		C1_04880C	*MRPL37*	C7_00950W	*YML6*	C3_02030W		C4_04820C	
C7_01440W		CR_01390W	*MGE1*	C3_05440C		C7_02120C		C7_01020C	
CR_10120C									
Cellular transport (5)									
CR_02920C	*AQY1*	C6_03790C	*HGT10*	C2_01020W	*HGT6*	C6_04610C	*NAG3*	C6_03390W	
Transcription (11)									
C4_05880W	*GAT1*	C2_00280C		CR_10690W	*POP3*	CR_02030C		CR_01710W	
C2_09460C		C2_05230C	*RPF2*	C5_01480W	*FYV5*	C6_02910W	*POP4*	C6_01040C	
C5_00980W	*TRY3*								
Cell wall biogenesis (3)									
CR_04420C	*RBR2*	CR_01930C	*BIO2*	C4_00720W	*CSP2*				
Unknown (20)									
CR_09350C		C2_06440C		C6_00720C	*COX15*	C5_01785W		CR_06330C	
C1_11320C		C3_00120W		CR_06920W		C4_03300C		C1_00970W	
C3_03490W	*RSN1*	C3_04510W		C1_04600C		C1_14480W		C3_00410C	
C4_06960W		C1_10500W		C1_09820C		C7_03210W		C1_10250C	

Among the 219 genes positively regulated by CaCrz1, a total of 40 genes have also been identified by DNA microarray analysis in the previous study (Table 2; 28). Through the online MEME (Multiple Em for Motif Elicitation) software Suite 5.0.2 (http://meme-suite.org/), we scanned promoters of these shared 40 genes and identified a consensus sequence [5′-GGAGGC(G/A)C(T/A)G-3′], which is different from the putative CaCrz1-binding consensus sequence [5′-G(C/T)GGT-3′] previously identified through DNA microarray [28], but similar to *S. cerevisiae* ScCrz1-binding motif [5′-GNGGC(G/T)CA-3′] [48]. Therefore, CaCrz1 can bind to two different CaCrz1-binding motifs in promoters of its target genes. This has also been reported previously for *M. oryzae* MoCrz1 [49, 50].

### CaCrz1 binds in vitro and in vivo to two putative binding motifs in the promoter of *CaUTR2*

**Fig. 3:**
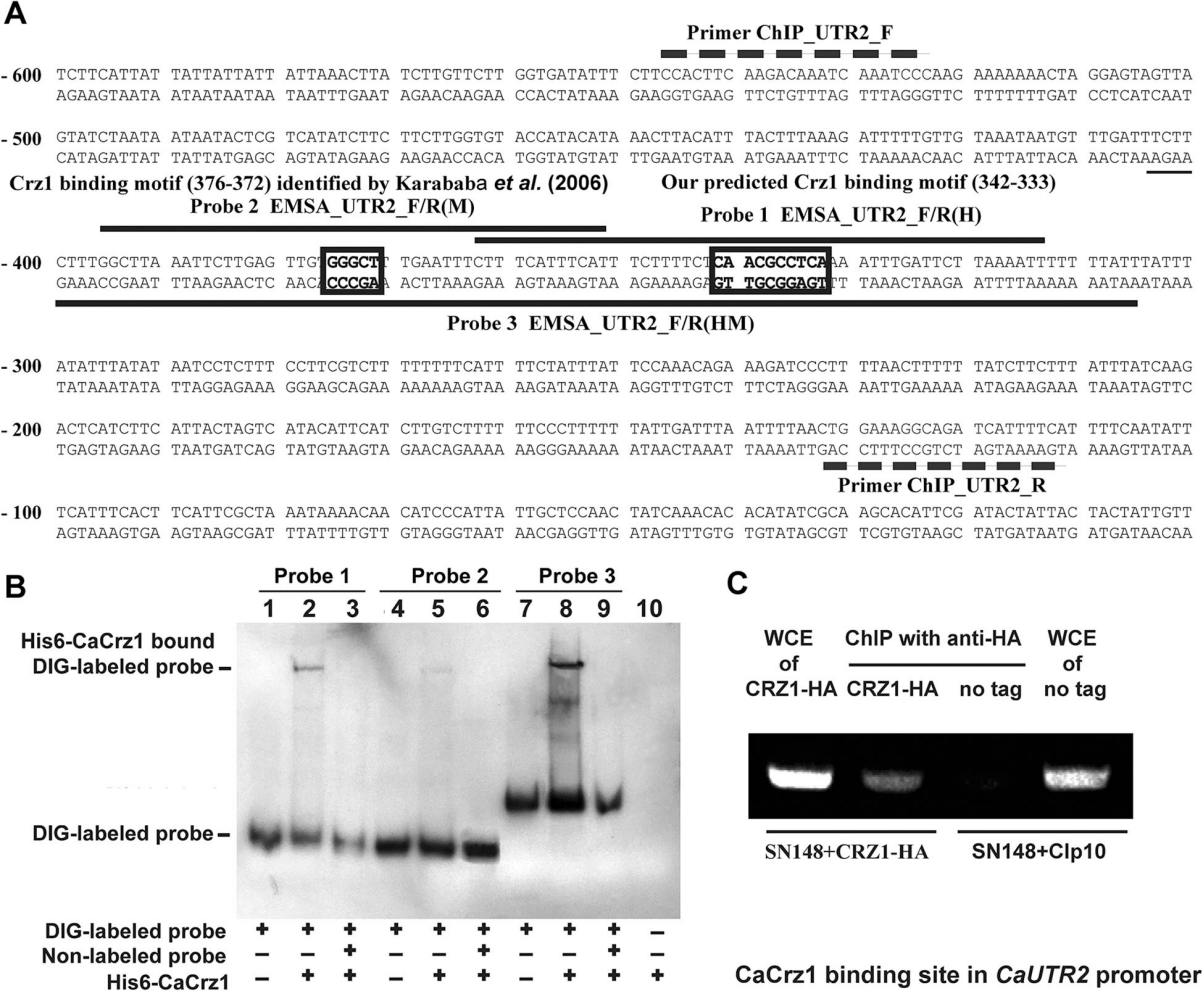
**CaCrz1 binds in vitro and in vivo to two motifs in the promoter of** ***UTR2*.** (**a**) Locations of two potential Crz1-binding motifs (boxed) in the *UTR2* promoter. The 5′-TGAGGCGTTG-3′ region in the complementary sequence of the 5′-C(− 342)AACGCCTCA(− 333)-3′ site is the potential Crz1 binding motif predicted in our study, and the 5′-G(− 376)GGCT(− 372)-3′ region is the putative Crz1 binding motif identified previously (28). Locations of EMSA Probe 1 [EMSA_UTR2_F/R(H)] and Probe 2 [EMSA_UTR2_F/R(M)] are indicated with dark lines above their corresponding sequences, and EMSA Probe 3 [EMSA_UTR2_F/R(HM)] is indicated with a dark line under its corresponding sequence. Locations of the ChIP PCR primer pair [CHIP_UTR2_F和CHIP_UTR2_R] are indicated with broken lines above and under their corresponding sequences, respectively. (**b**) DIG-labelled probe 1 [EMSA_UTR2_F/R (H)] was added into samples in Lanes 1–3. DIG-labelled probe 2 [EMSA_UTR2_F/R(M)] was added into samples in Lanes 4–6. DIG-labelled probe 3 [EMSA_UTR2_ F/R(HM)] was added into samples in Lanes 7–9. Purified His6-Crz1 protein of 1 μg was added into Lanes 2, 3, 5, 6, 8 and 9. Unlabelled probes 1, 2 and 3 were added into samples in Lanes 3, 6 and 9, respectively. Only purified His6-Crz1 protein, but not probe DNA, were added into the sample in Lane 10. (**c**) Detection of Crz1 binding to the *UTR2* promoter in vivo by ChIP analysis. The wild-type strain expressing Crz1-HA and the control strain integrated with CIp10 vector (no tag control) were exposed to 0.2 M CaCl_2_ for 1 h, and their cells were treated with formaldehyde. Whole cell extractions were obtained from collected cells, and immunoprecipitation was done with anti-HA monoclonal antibodies. Immunoprecipitated pellets were used as templates for PCR with the primer pair ChIP_UTR2_F/R. PCR products were separated on 1% agarose gel

Base on the consensus motif [5′-GGAGGC(G/A)C(T/A)G-3′] from the MEME analysis described above, we found one putative CaCrz1 binding motif, the 5′-TGAGGCGTTG-3′ region in the complementary sequence of the 5′-C(− 342)AACGCCTCA(− 333)-3′ site in the promoter of one of the CaCrz1 target genes, *CaUTR2* (Fig. 3a). Next, we tested the roles of this motif and the other putative CaCrz1 binding motif, 5′-G(− 376)GGCT(− 372)-3′, which was identified previously [28].

Different from other eukaryotes, *C. albicans* does not follow the universal genetic code, by translating the CTG codon into serine instead of leucine [51]. Therefore, we first optimized the codon usage by mutating all five CTG codons in *CaCRZ1* to TCT codon (L22S), AGC codon (L24S), TCC codons (L601S, L649S and L686S) (Additional file 1: Figure S2). The codon-optimized and Hisx6 tagged full-length CaCrz1 (His6-CaCrz1) was expressed in bacterial cells and purified (Additional file 1: Figure S3). Electrophoretic mobility shift (EMSA) assay showed that His6-CaCrz1 bound to both the P1 probe containing the putative binding motif identified in our study (Lane 2), the P2 probe containing the putative binding motif identified in the previous study [28] (Lane 5), and the Probe 3 containing two of the motifs (Lanes 8) (Fig. 3b). The binding of His6-CaCrz1 to Probe 1, Probe 2 and Probe 3 was abolished by their specific competitors, unlabelled probes, respectively (Lanes 3, 6 and 9) (Fig. 3b). Taken together, these results demonstrate that CaCrz1 can indeed bind in vitro to both motifs in the *CaUTR2* promoter.

To examine if CaCrz1 binds to the *CaUTR2* promoter region in vivo, we carried out chromatin immunoprecipitation (ChIP) experiments. We examined the wild-type SN148 strain expressing a chromosomally and C-terminally HA-tagged CaCrz1 (CaCrz1-HA) under the control of the *CaCRZ1* promoter (left two lanes in Fig. 3c), and the wild-type SN148 strain with the untagged wild type CaCrz1 and with the CIp10 vector integrated as the control (right two lanes in Fig. 3c). DNA samples isolated from their anti-HA chromatin immunoprecipitates were used in PCR assays to detect CaCrz1-HA target promoters (The second and the third lanes in Fig. 3c). As controls, their whole-cell extracts (WCEs) were used in parallel PCR assays to ensure the equivalence of the IP starting materials (The first and the fourth lanes in Fig. 3c). We found that the promoter region containing two putative binding motifs in the *CaUTR2* promoter were enriched in the anti-HA IPs of the CaCrz1-HA strain (The second lane in Fig. 3c), but not in the untagged CaCrz1 strain (The third lane in Fig. 3C). Together, these data demonstrate that CaCrz1 binds in vivo to the promoter region containing the two motifs of *CaUTR2*.

### Mutations of two putative binding motifs in the promoter abolish the CaCrz1-regulated expression of *CaUTR2*

**Fig. 4:**
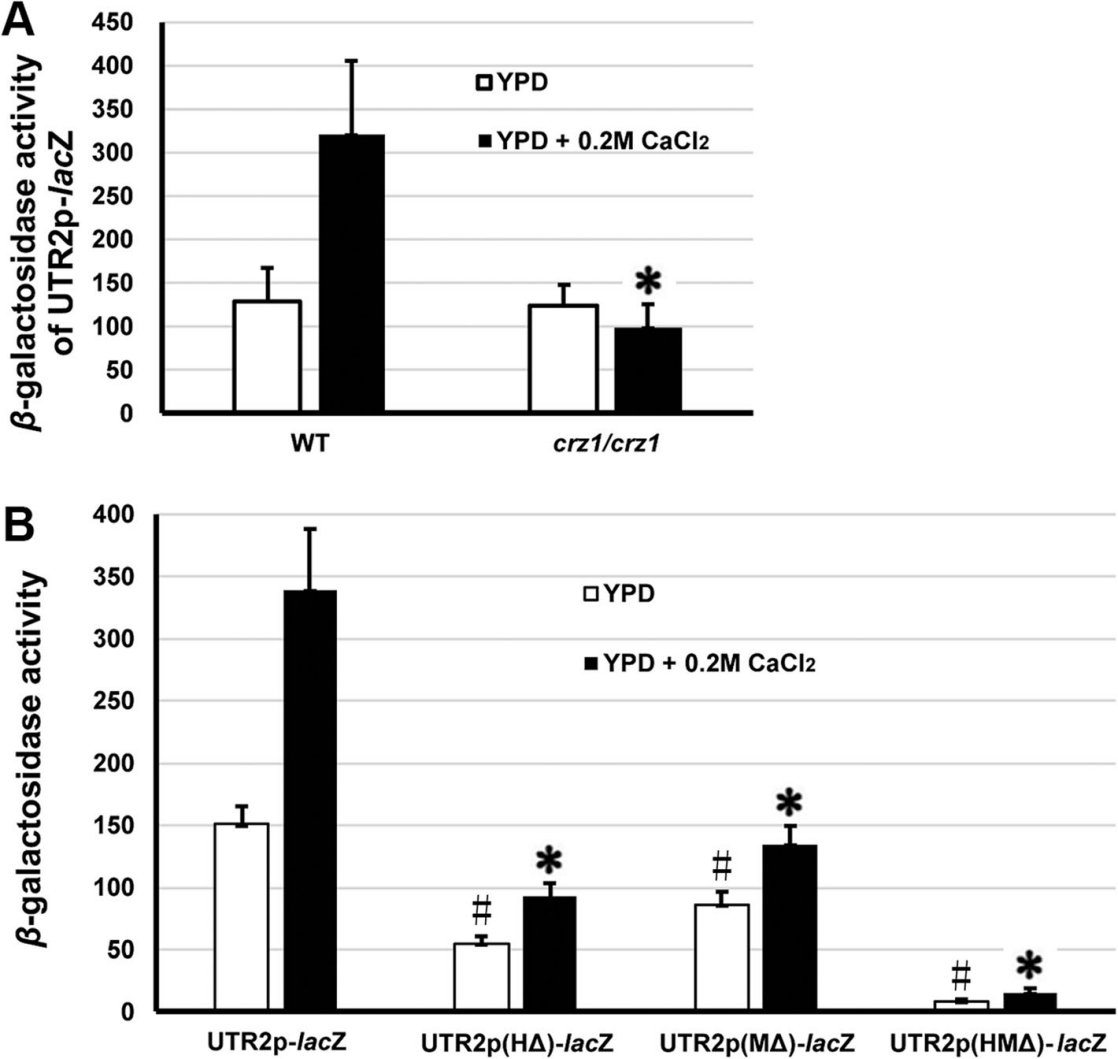
**Two CaCrz1-binding motifs in the promoter play additive roles in the regulation of** ***CaUTR2*** **expression.** (**a**), β-galactosidase activities of the wild-type promoter UTR2-lacZ in the wild-type SN148 and the *crz1/crz1* mutant cells in the absence or presence of 0.2 M CaCl_2_. The asterisk (*) indicates statistically significant differences (*P* < 0.05) in the β-galactosidase activity between the wild type strain SN148 and the *crz1/crz1* mutant strain in the absence or presence of 0.2 M CaCl_2_, respectively. (**b**), β-galactosidase activities of the wild-type promoter UTR2-lacZ, two single mutated promoters UTR2(HΔ)-lacZ and UTR2(MΔ)-lacZ as well as the double mutated promoter UTR2(HMΔ)-lacZ in the wild-type SN148 cells in the absence or presence of 0.2 M CaCl_2_. The asterisks (^#^) and (*) indicate statistically significant differences (P < 0.05) in the β-galactosidase activity between the wild type promoter and each of the mutated promoters in the wild-type strain SN148 in the absence or presence of 0.2 M CaCl_2_, respectively

To further characterize the effects of two CaCrz1-binding motifs on the expression of *CaUTR2*, we generated four plasmids, CIp10-UTR2-lacZ, CIp10-UTR2(HΔ)-lacZ, CIp10-UTR2(MΔ)-lacZ and CIp10-UTR2(HMΔ)-lacZ, containing the wild-type *CaUTR2* promoter, the single-motif mutated promoter UTR2(HΔ), the single-motif mutated promoter UTR2(MΔ) and the double-motif mutated promoter UTR2(HMΔ). In the absence of supplemented calcium, a basal expression level was detected for the wild type promoter UTR2-lacZ in the wild type cells (Fig. 4a). As expected, in response to 0.2 M CaCl_2,_ the β-galactosidase activity of the wild type promoter UTR2-lacZ was increased by more than two times in the wild-type cells, but did not change significantly in the *crz1/crz1* mutant cells (Fig. 4a). This indicates that the calcium-induced expression of *CaUTR2* is dependent on CaCrz1.

As compared to the wild-type promoter UTR2(HΔ), the β-galactosidase activities of two single mutated promoters UTR2(HΔ) and UTR2(MΔ) were significantly reduced in the absence or presence of 0.2 M CaCl_2_ in the wild type cells (Fig. 4b). The β-galactosidase activity of the double mutated promoter UTR2(HMΔ) were even further reduced than those of two single mutated promoters UTR2(HΔ) and UTR2(MΔ) in the absence or presence of 0.2 M CaCl_2_ in the wild type cells (Fig. 4b). Taken together, these results suggest that two CaCrz1-binding motifs play additive roles in the regulation of *CaUTR2* expression.

### CaCrz1 binds in vitro and in vivo to its own promoter

**Fig. 5:**
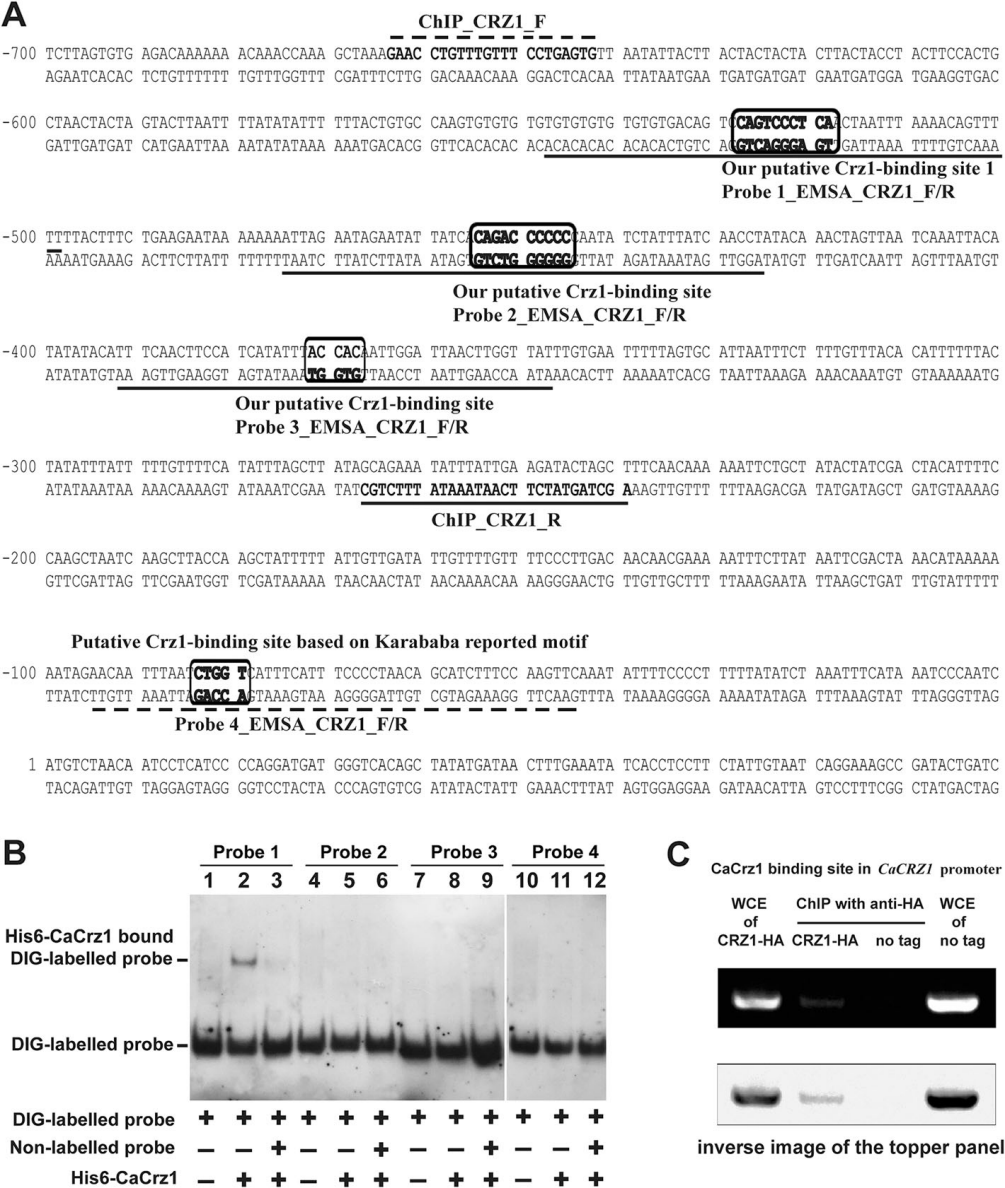
**CaCrz1 binds in vitro and in vivo to its own promoter.** (**a**) Locations of three predicated CaCrz1-binding motifs (boxed and within Probe 1, Probe 2 and Probe 3 sequences) based on the consensus motif we discovered in this study and one predicated CaCrz1-binding motif (boxed and within the Probe 4 sequence). Locations of the ChIP PCR primer pair [CHIP_CRZ1_F和CHIP_CRZ1_R] are indicated with broken lines above and under their corresponding sequences, respectively. (**b**) DIG-labelled Probe 1_EMSA_CRZ1_F/R was added into samples in Lanes 1–3. DIG-labelled Probe 2_EMSA_CRZ1_F/R was added into samples in Lanes 4–6. DIG-labelled Probe 3_EMSA_CRZ1_ F/R was added into samples in Lanes 7–9, and DIG-labelled Probe 4_EMSA_CRZ1_ F/R was added into samples in Lanes 10–12. Unlabelled probes 1, 2, 3 and 4 were added into samples in Lanes 3, 6, 9 and 12, respectively. Purified His6-Crz1 protein of 1 μg was added into Lanes 2, 3, 5, 6, 8, 9, 11 and 12. (**C**) Detection of CaCrz1 binding to its own promoter in vivo by ChIP analysis. The same pair of strains were treated and their whole cell extracts were immunoprecipitated as in Fig. 3c. PCR reactions were carried out with ChIP primers CHIP_CRZ1_F和CHIP_CRZ1_R. The lower panel is the inverse image of the topper panel, which is for a better view of the PCR band in the second lane

Both a previous study and our current study have observed that *CaCRZ1* itself is positively regulated by CaCrz1 (Table 2; 28). Base on the consensus motif [5′-GGAGGC(G/A)C(T/A)G-3′] identified in our study, we identified two putative CaCrz1 binding motif, the 5′-T(− 519)GAGGGACTG(− 528)-3′ site (within the Probe 1 sequence) and the 5′-G(− 446)GGGGGTCTG(− 455)-3′ site (within the Probe 2 sequence) in the complementary sequence, in its own promoter (Fig. 5a). Based on the consensus motif [5′-G(C/T)GGT-3′] identified previously [28], we also identified one putative CaCrz1 binding motif, the 5′-G(− 368)TGGT(− 372)-3′ site (within the Probe 3 sequence), in the complementary sequence of *CaCRZ1* promoter (Fig. 5a). The fourth putative CaCrz1 binding motif, the 5′-C(− 84)TGGT(− 80)-3′ site (within the Probe 4 sequence) was identified previously [28].

EMSA assay demonstrated that His6-CaCrz1 bound to only the P1 probe (Lane 2), but not to other three probes, Probe 2 (Lane 5), Probe 3 (Lane 8) and Probe 4 (Lane 11) (Fig. 5b). The binding of His6-CaCrz1 to Probe 1 was abolished by its specific competitor, unlabelled Probe 1 (Lane 3) (Fig. 5b). ChIP analysis indicated that the promoter region containing the 5′-T(− 519)GAGGGACTG(− 528)-3′ site (within the Probe 1 sequence) was enriched in the anti-HA IPs of the CaCrz1-HA strain (Lane 2), but not in the untagged CaCrz1 strain (lane 3) (Fig. 5c). These results demonstrate that CaCrz1 regulates its own expression by binding to the motif 5′-T(− 519)GAGGGACTG(− 528)-3′ in its own promoter. The autoregulation phenomenon of this transcription factor gene has also been previously shown in the rice blast pathogen *M. oryzae* MoCrz1 [49, 50].

## Discussion

Microarrays are based on the hybridization of oligonucleotide DNA sequences, representing the entire set of genes of an organism arranged in a grid pattern, with complementary DNA (cDNA) molecules derived from the transcriptome in a cell sample, while cDNA molecules derived from a sample are directly and massively sequenced in the case of RNA-sequencing methodology [52, 53]. As compared to microarrays, RNA sequencing technology offers increased specificity and sensitivity, but the application of multiple transcriptome measurement methods can improve the comprehension of the global gene expression profile of one organism [34, 35]. Through RNA sequencing, we have identified 219 genes positively, and 59 genes negatively, regulated by CaCrz1 in response to calcium stress in *C. albicans*. A total of 40 out of the 219 genes identified in this study to be positively regulated by CaCrz1 account for the majority of 60 genes identified by DNA microarray analysis in the previous study (Table 2; 28). Therefore, our current study has expanded the global expression profile of genes controlled by CaCrz1 in response to calcium stress in *C. albicans*. This provides a basis for further understanding the regulation of calcium homeostasis in this important human fungal pathogen.

**Fig. 6:**
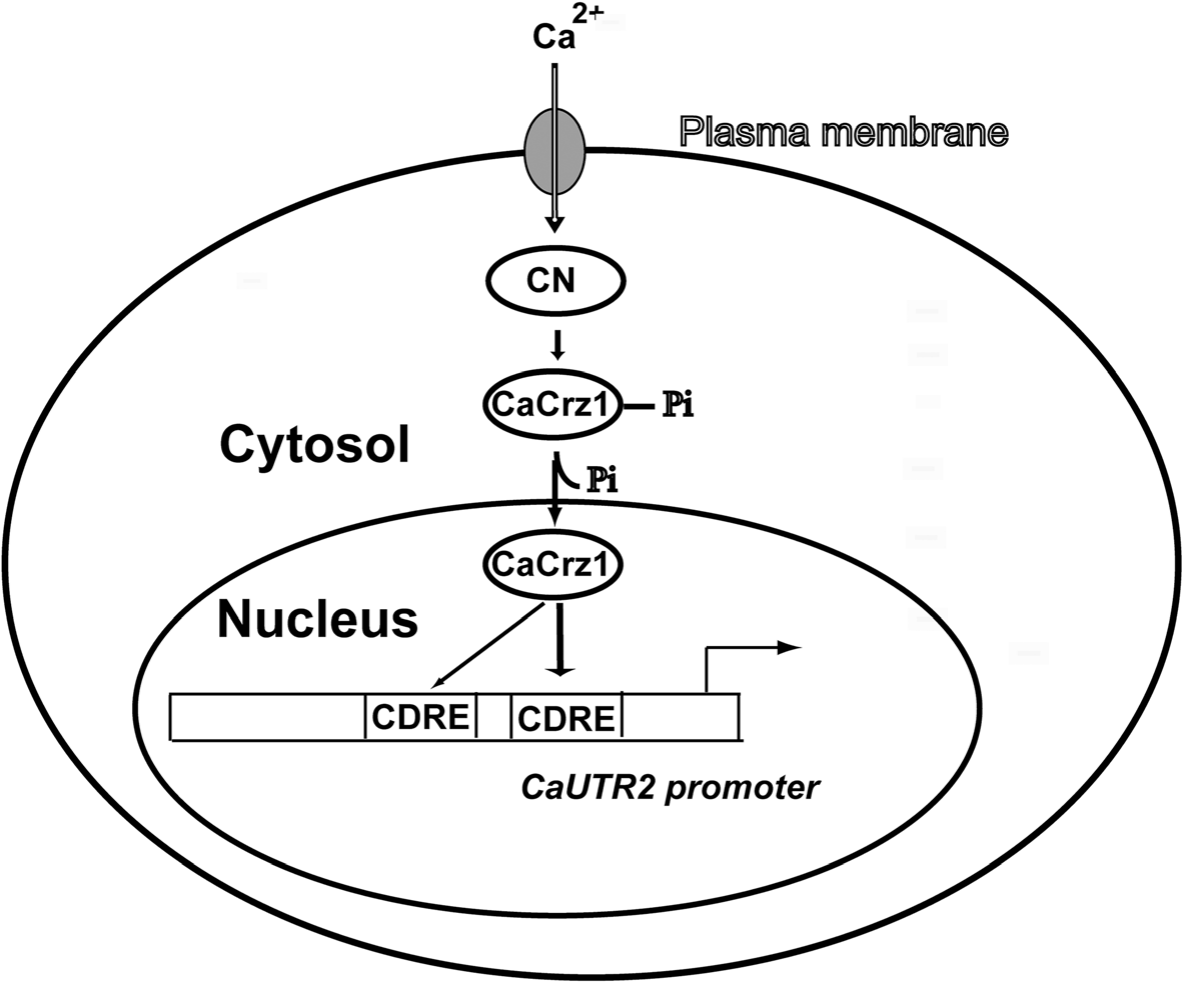
**Schematic model for the regulation of** ***CaUTR2*** **expression by the transcription factor CaCrz1 in response of** ***C. albicans*** **cells to extracellular calcium stress.** Influx of extracellular calcium ions to the cytosol leads to the activation of calcineurin, which in turn dephosphorylates and activates CaCrz1. Dephosphorylated CaCrz1 enters to the nucleus to bind to two CaCrz1 binding motifs (calcineurin dependent response element; CDRE) in the promoter of *CaUTR2*, which results in the activation of *CaUTR2* expression

In addition to the CaCrz1-binding motif (M) identified in the previous study [28], we have revealed a novel CaCrz1-binding motif (H) through the MEME analysis of promoters of 40 common genes identified to be controlled by CzCrz1 through both RNA sequencing and microarray approaches (Fig. 3). Furthermore, we have demonstrated that CaCrz1 binds in vitro and in vivo to these two motifs in the promoter of its target gene *CaUTR2*, and that these two calcineurin-dependent response elements (CDREs) might play additive roles in the regulation of *CaUTR2* expression (Fig. 6). Similarly, two MoCrz1-binding motifs in promoters of target genes have been demonstrated in the rice fungal pathogen *M. oryzae* [49]. Among the 219 genes positively regulated by CaCrz1, we found that promoters of 79 genes contain both motifs (M and H), promoters of 59 genes contain only motif H, promoters of 45 genes contain only motif M, and promoters of 36 genes contain neither motif H or motif M (Additional file 2). Therefore, expression of target genes seems to be differentially regulated by CaCrz1.

In *S. cerevisiae*, 125 calcium-specific and calcineurin-dependent genes reported in a previous study [48]. Out of these 125 genes, there are 83 genes that are positively regulated by ScCrz1 (Additional file 3). From the *C. albicans* database (http://www.candidagenome.org/), we were able to find 38 *C. albicans* homologs for these ScCrz1-dependent *S. cerevisiae* genes, but only 9 of these 38 *C. albicans* homologs are present in the list of genes identified in this study to be CaCrz1-dependent (Table 2; Additional file 3). Therefore, target genes of ScCrz1 and CaCrz1 seem to be very divergent. This is supported by our observation that the amino acid sequences of ScCrz1 and CaCrz1 shares only 31.9 and 24% similarity and identity, respectively, although their predicted structures are very similar (Fig. S4 in Additional file 1). Similar to the homologs in *S. cerevisiae*, *M. oryzae* and another human fungal pathogen *Aspergillus fumigatus* [49], expression of *PMC1* (C3_01250W_A) and *RCT1* (C3_05710W) is positively controlled by CaCrz1, although expression of *RCN1* (C6_01160W_A) is not regulated by CaCrz1 (SuppInfo 1 and 2; GEO Accession number: GSE123122). This is consistent with previous observations on *Cryptococcus neoformans CBP1*, the homolog of *RCN1*, that neither is regulated by nor interacts with Crz1 in this human fungal pathogen [54, 55]. In contrast, expression of *RCN1* is regulated by Crz1 in *S. cerevisiae*, *M. oryzae* and another human fungal pathogen *Aspergillus fumigatus*, which forms a feedback mechanism for the regulatory role of Rcn1 as an inhibitor of calcineurin [48, 55, 56]. Nevertheless, overexpression of *C. albicans RCN1* could inhibit *S. cerevisiae* calcineurin function [21]. Taken together, these data indicate that regulation of the calcium/calcineurin signaling pathway is diverged in fungal pathogens, although the core calcium signaling machinery (calmodulin, calcineurin and Crz1) is highly conserved across these species. This is consistent with the previous hypothesis [49, 56, 57].

It is interesting to note that the calcium-sensitive phenotype of the CRISPR mutant for *CaCRZ1* could only be partially reversed by the introduction of the full-length *CaCRZ1* gene back to its genome (Fig. 1). Transcripts of the CRISPR mutant *CaCRZ1* from the *CaCRZ1* locus might compete with those of the wild-type *CaCRZ1* transcripts derived from CIp10-CaCRZ1 at the *CaRPS1* locus, which might interfere with the translational efficiency of wild-type *CaCRZ1* transcripts. This might explain the partial complementation of calcium sensitivity of the CRISPR mutant for *CaCRZ1* by CIp10-CaCRZ1. Furthermore, the full-length 6xHis tagged CaCrz1 protein expresses in bacterial cells as a protein of about 100 kDa (Additional file 1: Figure S3), which is much bigger than its predicted size (= 80 kDa). However, the dephosphorylated form of CaCrz1 expressed in *C. albicans* cells in response to calcium stress also shows a molecular weight of more than 100 kDa (Fig. 2), which is similar to that of CaCrz1 expressed in bacterial cells. Therefore, this mobility shift could be due to the conformation of CaCrz1 itself, but not the host cell environment or the tag type or tag location (N-terminus or C-terminus).

## Conclusions

In this study, through RNA sequencing we have identified 219 genes that are positively, and 59 genes that are negatively, controlled by CaCrz1 in response to calcium stress. We have also revealed and demonstrated experimentally a novel consensus CaCrz1-binding motif [5′-GGAGGC(G/A)C(T/A)G-3′] in promoters of CaCrz1 target genes. In addition, CaCrz1 binds to its own promoter and shows an autoregulatory mechanism for *CaCRZ1* expression. These findings would contribute to our further understanding of molecular mechanisms regulating calcium homeostasis.

## Supplementary information

**Additional file 2: Figure S1.** Construction and phenotypes of CRISPR mutants for *CaCRZ1.* **Figure S2.** Alignment between the amino acid sequences of the wild type and the codon optimized version of CaCrz1. **Figure S3.** Expression and purification of the codon optimized and His6-tagged CaCrz1 protein in bacterial cells. **Figure S4.** Differences between CaCrz1 and ScCrz1. **Table S1.** Primers used in this study.

**Additional file 3.** Promoter analysis of 219 genes whose expression is positively regulated by CaCrz1#

**Additional file 4. **Comparison of calcium-specific and Crz1-dependent genes in *Saccharomyces cerevisiae* and *Candida albicans.*

## Data Availability

All data generated or analyzed during this study are included in this published article and deposited in the database Gene Expression Omnibus (*GEO*) site.

## Abbreviations

ChIP: Chromatin immunoprecipitation
CRISPR: Clustered regularly interspaced short palindromic repeat
Crz1: Calcineurin-responsive zinc finger 1
EMSA: Gel electrophoretic mobility shift assay
MEME: Multiple em for motif elicitation
NFAT: the nuclear factor of activated T cells
PCR: Polymerase chain reaction
YPD: Yeast peptone dextron
