# Supplementary material for: RNA sequencing reveals an additional Crz1-binding motif in promoters of its target genes in the human fungal pathogen Candida albicans
Source: Cell Commun Signal. 2020 Jan 3;18:1. doi: 10.1186/s12964-019-0473-9 (PMC6942403; doi:10.1186/s12964-019-0473-9)
Supplement: Supplementary file 2 — Additional file 2: Figure S1. Construction and phenotypes of CRISPR mutants for CaCRZ1. Figure S2. Alignment between the amino acid sequences of the wild type and the codon optimized version of CaCrz1. Figure S3. Expression and purification of the codon optimized and His6-tagged CaCrz1 protein in bacterial cells. Figure S4. Differences between CaCrz1 and ScCrz1. Table S1. Primers used in this study. [file 12964_2019_473_MOESM2_ESM.pdf]

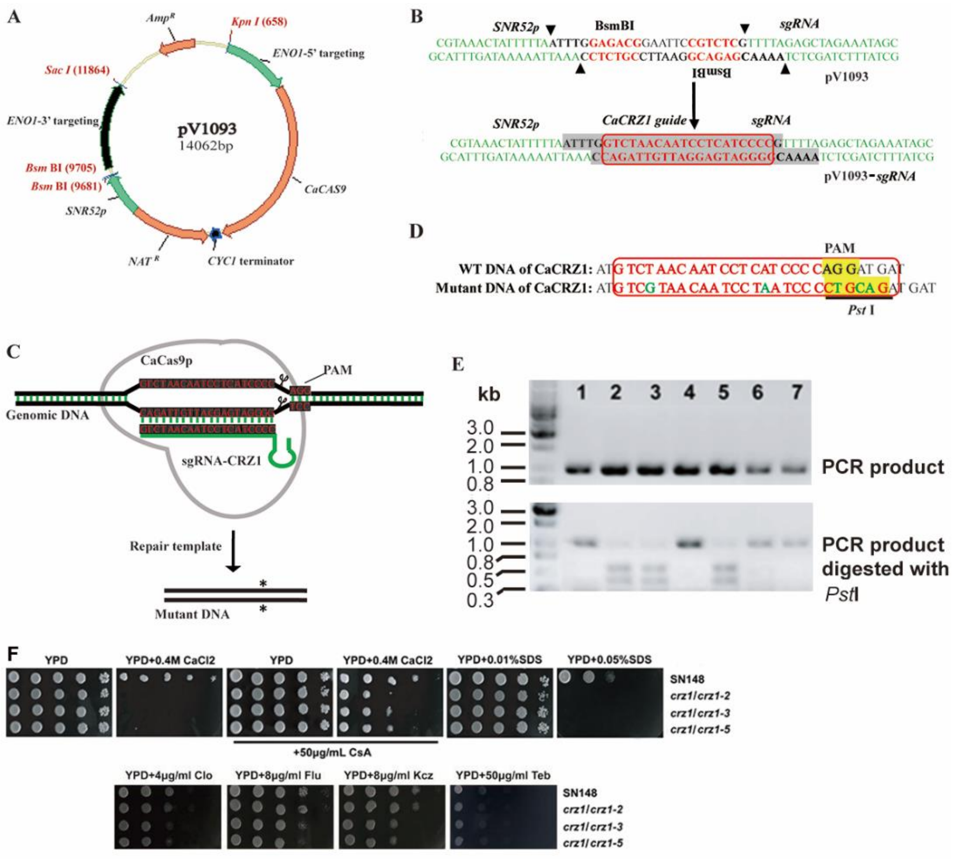

Figure S1. Construction and phenotypes of CRISPR mutants for *CaCRZ1*. A, The plasmid pV1093 contains *CaCAS9* for genome-editing, the antibiotic nourseothricin (NAT) resistant gene (NAT^{R}) for selection of *C. albicans* transformants, the ampicillin resistant gene Amp^{R} for selection of *E. coli* transformants, and the flanking sequences of *CaENO1* (ENO1-5' targeting and ENO1-3' targeting). The flanking sequences of *CaENO1* are used for targeting the *CaENO1* locus for integration of pV1093 or pV1093-SgRNA [58]. **B**, the sequence of *CaCRZ1* synthetic guide RNA (sgRNA) (in red bracket) in the pV1093-sgRNA recombinant plasmid. **C**, Schematic of Cas9 mutagenesis of *CaCRZ1*. The system simultaneously creates homozygous mutations (*) in the protospacer adjacent motif (PAM) site (**D**) of the *CaCRZ1* to prevent repeated cleavage subsequent to integration. **D**, Two stop codons (TAA) and one *Pst*I restriction site are introduced around the PAM site of the mutated *crz1* allele. **E**, Diagnostic *Pst*I digestion of PCR products derived from genomic DNA samples of seven *C. albicans* transformants integrated with both repair DNA and pV1093-SgRNA plasmid DNA linearized by both *Sac*I and *Kpn*I. PCR products were amplified with primers CRZ1-CF and CRZ1-CR (top panel), and they were digested with *Pst*I (bottom panel). Three independent *C. albicans* transformants No. 2 (HHCA184), No. 3 (HHCA185) and No. 5 (HHCA187) were potential correct CRISPR mutants for *CaCRZ1*, whose mutated sites in *CaCRZ1* alleles were further confirmed by DNA sequencing. DNA samples were run on 1% agarose gel. **F**, Phenotypes of CRISPR mutants for *CRZ1*. The wild type SN148 and the three CRISPR mutants for *CRZ1* were grown overnight in YPD medium, and their overnight cultures were serially diluted by 10 times and spotted on plates indicated. Plates were incubated at 30°C for 2-3 days. CsA, cyclosporine A. Clo, clotrimazole . Flu, fluconazole. Kcz, ketoconazole. Teb, terbinafine.

Sequence	Start	Alignment
		1 ... 50
WT of CaCRZ1	(1)	MSNNPHPQDDGSQLYDNFEISPPSIVIRKADTDQSLNKIMLNQESQDINN
Optimized version of CaCRZ1	(1)	MSNNPHPQDDGSQLYDNFEISPPSIVIRKADTDQSLNKIMLNQESQDINN
		51 ... 100
WT of CaCRZ1	(51)	YYTENVKNDNNPNNSYQDYTFSGNSSNQQHQQQQQQHLYEDLPTQFHYSN
Optimized version of CaCRZ1	(51)	YYTENVKNDNNPNNSYQDYTFSGNSSNQQHQQQQQQHLYEDLPTQFHYSN
		101 ... 150
WT of CaCRZ1	(101)	NSFFEPPPAPTVELTDDPLPNFNYPPSNIYINDNASDISLNTKDLPQFTT
Optimized version of CaCRZ1	(101)	NSFFEPPPAPTVELTDDPLPNFNYPPSNIYINDNASDISLNTKDLPQFTT
		151 ... 200
WT of CaCRZ1	(151)	NEFLSPTSQLSTPFSPGHYSQ**LL**QDFLQVNHTNGSGNNNNNNNNNNLLNP
Optimized version of CaCRZ1	(151)	NEFLSPTSQLSTPFSPGHYSQ**SS**QDFLQVNHTNGSGNNNNNNNNNNLLNP
		201 ... 250
WT of CaCRZ1	(201)	RSPSQYSSHSLYSDNSSQPASPFLDAASHVSNNSFIPPVIPTALSDVGSQ
Optimized version of CaCRZ1	(201)	RSPSQYSSHSLYSDNSSQPASPFLDAASHVSNNSFIPPVIPTALSDVGSQ
		251 ... 300
WT of CaCRZ1	(251)	NLDPSHNLGLSANQHFDSVNEFLSTGEIQLGQSVSSTNLPSMEEDSIKWG
Optimized version of CaCRZ1	(251)	NLDPSHNLGLSANQHFDSVNEFLSTGEIQLGQSVSSTNLPSMEEDSIKWG
		301 ... 350
WT of CaCRZ1	(301)	GGNGQEAYTSLAMMEQRASADNSGMRLATHQFSETQIKQEDQQTNMNHQY
Optimized version of CaCRZ1	(301)	GGNGQEAYTSLAMMEQRASADNSGMRLATHQFSETQIKQEDQQTNMNHQY
		351 ... 400
WT of CaCRZ1	(351)	TFSNPQMNFDFDITVTPPPQQLEVKPFGNDKDMNNSSGTTNNNNNNSQFD
Optimized version of CaCRZ1	(351)	TFSNPQMNFDFDITVTPPPQQLEVKPFGNDKDMNNSSGTTNNNNNNSQFD
		401 ... 450
WT of CaCRZ1	(401)	IVSTAATNNSNQLLTENNLSNYNQLQRTEQGNDNDSLQIHRDATGIIISI
Optimized version of CaCRZ1	(401)	IVSTAATNNSNQLLTENNLSNYNQLQRTEQGNDNDSLQIHRDATGIIISI
		451 ... 500
WT of CaCRZ1	(451)	NQAPEEIAAKTPSLFSNSSANSSIHNSPRSDIDNKSGQYYNNGGDGNSLV
Optimized version of CaCRZ1	(451)	NQAPEEIAAKTPSLFSNSSANSSIHNSPRSDIDNKSGQYYNNGGDGNSLV
		501 ... 550
WT of CaCRZ1	(501)	PNSQLLPSSPNSNNDNYGGGGSSNDENNLLNPEEFQSVKRGRRKSHASRT
Optimized version of CaCRZ1	(501)	PNSQLLPSSPNSNNDNYGGGGSSNDENNLLNPEEFQSVKRGRRKSHASRT
		551 ... 600
WT of CaCRZ1	(551)	STNPNSLSPRSRSRSRSSAKSSNDAVISDNDESDDVLQSREKMLELALPS
Optimized version of CaCRZ1	(551)	STNPNSLSPRSRSRSRSSAKSSNDAVISDNDESDDVLQSREKMLELALPS
		601 ... 650

```
                  WT of CaCRZ1  (601) LSSKRTQKHPSLYACHLCDKRFTRPYNLKSHIRTHTQEKPFICSKCGKLF
Optimized version of CaCRZ1  (601) SSSKRTQKHPSLYACHLCDKRFTRPYNLKSHIRTHTQEKPFICSKCGKSF
                             651                                          700
                  WT of CaCRZ1  (651) ARSHDKKRHELLHQGIKNFKCEGYLQDGTRWGCGKLFARADALRRHFQTE
Optimized version of CaCRZ1  (651) ARSHDKKRHELLHQGIKNFKCEGYLQDGTRWGCGKSFARADALRRHFQTE
                             701                           731
                  WT of CaCRZ1  (701) AGKQCVKRLLLEEQANSSGKPLATSSGVEIT-
Optimized version of CaCRZ1  (701) AGKQCVKRLLLEEQANSSGKPLATSSGVEIT-
```

Figure S2. Alignment between the amino acid sequences of the wild type and the codon optimized version of CaCrz1. All five CTG. codons (L22, L23, L601, L649 and L686) in the wild type are mutated to TCT codon (L22S), AGC codon (L24S), TCC codons (L601S, L649S and L686S) in the optimized version.

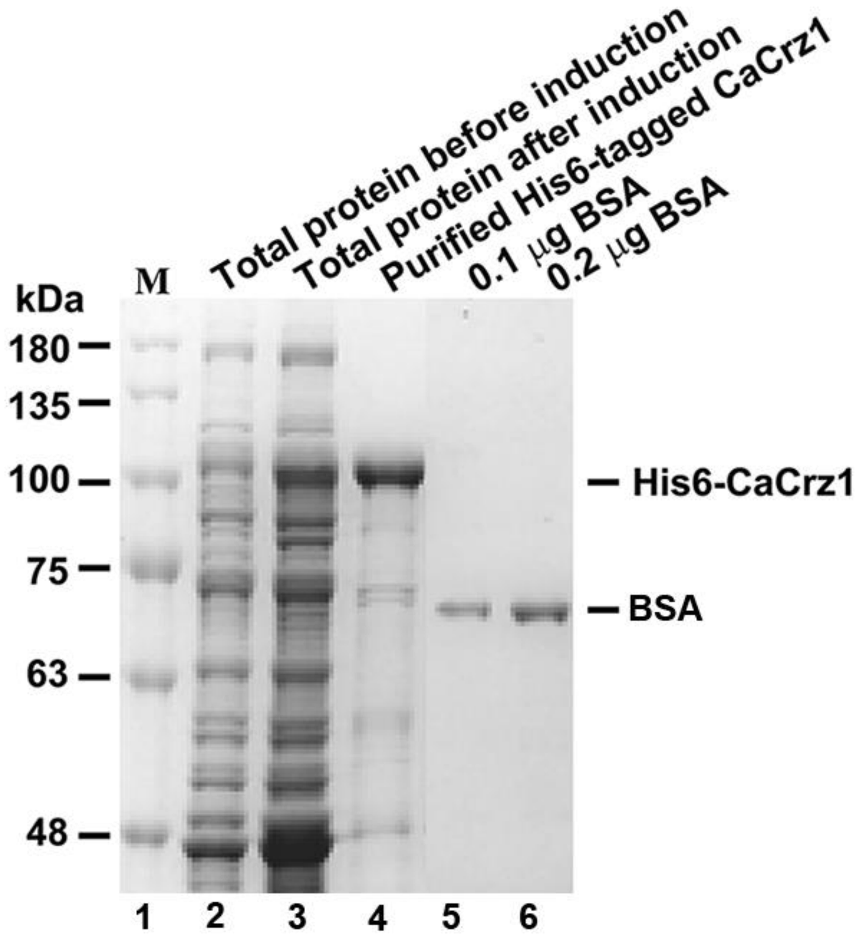

Figure S3. Expression and purification of the codon optimized and His6-tagged CaCrz1 protein in bacterial cells. Detection of the IPTG-induced and the purified His6-CaCrz1 fusion protein by Coomassie blue staining. Lane 1, protein size maker. Lane 2, the total protein from *E. coli* cells containing pET28-CaCrz1 before induction by IPTG. Lanes 3, the total proteins from *E. coli* cells induced by IPTG. Lanes 4, the purified codon optimized and His6-tagged Crz1 proteins. Lanes 5 and 6, 0.1 μg and 0.2 μg BSA protein, respectively.

A

```
CaCrz1     (1) MSNNPHPQDDGSQLYDNFEISPPSIVIRKADTDQSLNKIMLNQESQDINNYYTENVKNDNNPNNSYQDYTFSGNSSNQQHQQQQQQHLYEDLPTQFHYSN
ScCrz1     (1) --------------------------MSFSNGNMASYMTSSNGEEQSINN------KNDIDDNSAYRRNNFR-NSSNSGSHTFQLSDLDLDVDMRMDSAN

CaCrz1   (101) NS-FFEPPPAPTVELTDDPLPNFNYPPSNIYINDNASDISLNTKDLPQFTTN--------EFLSPTSQLSTPFSPGHYSQSSQDFLQVNHTNGSGNNNNN
ScCrz1    (68) SSEKISKNLSSGIPDSFDSNVNSLLSPSSGSYSADLNYQSLYKPDLPQQQLQQQQLQQQQQQQQQQQQQQKQTPTLKVEQSDTFQWDDILTPADNQHRP

CaCrz1   (192) NNNNNLLNPRS--P---SQYSSHSLYSDNSSQPASPFLDAASHVSNNSFIPPVIPTALSDVGSQNLDPSHNLGLSANQHFDSVNEFLSTGEIQLGQSVSS
ScCrz1   (168) SLTNQFLSPRSNYDGTTRSSGIDSNYSDTESNYHTPYLYPQDLVSSPAMSHLTANNDDFDDLLSVASMNSNYLLPVNSHGYKHISNLDELDDLLSLTYSD

CaCrz1   (287) TNLPSMEEDSIKWGGGNGQEAYTSLAMMEQRASADNSGMRLATHQFSETQIKQEDQQTNMNHQYTFSNPQMNFDFDITVTPPPQQLEVKPFGNDKDMNNS
ScCrz1   (268) NNLLSASNNSDFNNSNNG---IINTADTQNSTIAINK-SKVGTNQKMLLTIPTSSTPSPSTHAAPVTPIISIQEFNEGHFPVKNEDDGTLQLKVRDNESY

CaCrz1   (387) SGTTNN--NN-NNSQFDIVSTAATNNSNQLLTENNLSNYNQLQRTEQGNDNDSLQIHRDATGIIISINQAPEEIAAKTPSLFSNSSANSSIHNSPRSDID
ScCrz1   (364) SATNNNNLLRPDDNDYNNEALSDIDRSFEDIINGRKLKLKKSRRRSSQTSNNSFTSRRSSRSRSISPDEKAKSISANREKLLEMADLLPSSEN------D

CaCrz1   (484) NKSGQYYNNGGDGNSLVPNSQLLPSSPNSNNDNYGGGGSSNDENNLLNPE-EFQSVKRGRRKSHASRTSTNPNSLSPRSRSRSRSSAKSSNDAVISDNDE
ScCrz1   (458) NNRERYDNDSKTSYNTINSS-------NFNEDNNN--------NNLLTSKPKIESGIVNIKNELDDTSKDLGILLDIDSLGQFEQKVGFKNDDNHENNDN

CaCrz1   (583) SDDVLQSREKMLELALPSSSSKRTQKHPSLYACHLCDKRFTRPYNLKSHIRTHTQEKPFICSKCGKSFARSHDKKRHELLHQGIKNFKCEGYLQDGTRWG
ScCrz1   (543) GTFSVKKNDNLEKLDSVTNN----RKNPANFACDVCGKKFTRPYNLKSHLRTHTNERPFICSICGKAFARQHDRKRHEDLHTGKKRYVCGGKLKDGKPWG

CaCrz1   (683) CGKSFARADALRRHFQTEAGKQCVKRLLLEEQANSSGKPLATSSGVEIT
ScCrz1   (639) CGKKFARSDALGRHFKTESGRRCITPLYEEARQEKSGQES---------
```

B

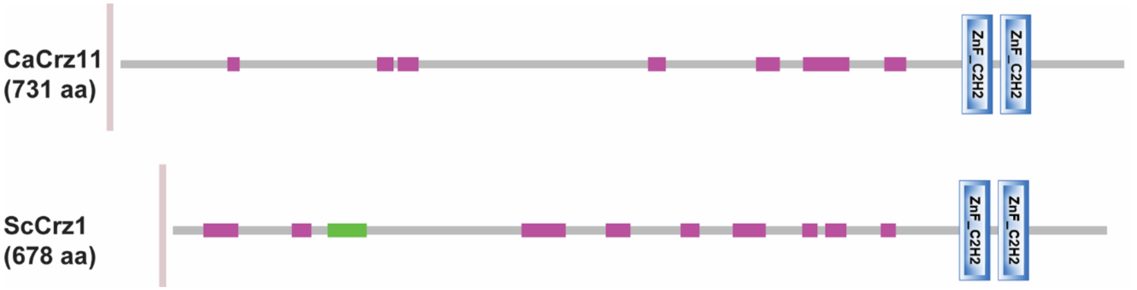

Figure S4. Differences between CaCrz1 and ScCrz1. (A) Amino acid sequence alignment between of CaCrz1 and ScCrz1. Their similarity and identity are 31.9% and 24%, respectively. All five CTG codons (L172S, L173S, L601S, L649S and L686S) have been converted according to *C. albicans* codon usage. **(B)** Predicted structures of CaCrz1 and ScCrz1 by the online SMART software (http://smart.embl-heidelberg.de/smart/set_mode.cgi?GENOMIC=1).

Table S1. Primers used in this study

Primer	Sequence
RZ1-sgF	atttgGTCTAACAATCCTCATCCCCg (*Bsm*BI site attached and underlined)
CRZ1-sgR	aaaacGGGGATGAGGATTGTTAGACc (*Bsm*BI site attached and underlined)
CRZ1-RF	tttccccttttatatctaaatttcataaatcccaatcatGTCG**TAA**CAATCC**TAA**TCCCCTGCAG (two introduced stop codons indicated in red and one introduced *Pst*I site is bolded)
CRZ1-RR	ggtgatatttcaaagttatcatatagctgtgacccatcatCTGCAGGGGA**TTA**GGATTG**TTA**CGAC (two introduced stop codons indicated in red and one introduced *Pst*I site is bolded)
CRZ1-CF	ACTGTGCCAAGTGTGTGTG
CRZ1-CR	GTGGTAAACTGAGGCAAGTCC
LR159F	CCAAGAAGCATCTAATCAACTCCC
LR159R	CGCGTCTAAGTTAGTCTCCGTTC
CRZ1-clonF	agggaacaaaagctgggtaccGTGTGTGGGTAGTGTATATTGTTCCA, bases in lower case from the *Kpn*I site (underlined) in the vector CIp10.
CRZ1-clonR	atcgataccgtcgacctcgagCGACTTGGCTACTTCTTTTTCTGC, bases in lower case from the *Xho*I site (underlined) in the vector CIp10.
lacZ_ORF_F(XhoI)	atcgataccgtcgacctcgagTCCATGAACATGACTGAAAAAATTCAA, the *Xho*I site is underlined
lacZ_ORF_R(KpnI)	agggaacaaaagctgggtaccCTAATTTAGTGGTTCAATCATGAAG, the *Kpn*I site is underlined
ACT1_T_F(KpnI)	gaaccactaaattagggtaccGAGTGAAATTCTGGAAATCTGGAA, the *Kpn*I site is underlined

ACT1_T_R(KpnI)	agggaacaaaagctgggtaccATTTTATGATGGAATGAATGGGATG, the *Kpn*I site is underlined
UTR2_P_F(XhoI)	atcgataccgtcgacctcgagGGAAACAGATCACACACGTACGA, the *Xho*I site is underlined
UTR2_P_R(XhoI)	ttcagtcatagccatctcgagAACAATAGTAGTAATAGTATCGAATGTGCTT, the *Xho*I site is underlined
UTR2_exF	tgaaggtttaaagatctgaatcg
UTR2_inR	aaatgctcgataatgttgcc
UTR2_(HΔ)_R	ttTCTAGAagaaaagaaatgaaatgaaagaaattcaa, the *Xba*I site is underlined
UTR2_(HΔ)_F	ttgaatttctttcatttcatttcttttctTCTAGAaaatttgattcttaaaattttttttattt, the *Xba*I site is underlined
UTR2_(MΔ)_R	agaaaagaaatgaaatgaaagaaattcaaTCTAGAacaactcaagaatttaagccaaag, the *Xba*I site is underlined
UTR2_(MΔ)_F	gtTCTAGAttgaatttctttcatttcatttc, the *Xba*I site is underlined
Probe_EMSA_CRZ1_F	AACAATTTAATCTGGTCATTTCATTTCCCCTAACAGCATCTTTCCAAGTT, putative CaCrz1-binding site is indicated in red.
Probe_EMSA_CRZ1_R	AACTTGGAAAGATGCTGTTAGGGGAAATGAAATGACCAGATTAAATTGTT
Probe1_EMSA_CRZ1_F	TGTGTGTGTGTGTGACAGTCCAGTCCCTCAACTAATTTAAAACAGTTTTT
Probe1_EMSA_CRZ1_R	AAAAACTGTTTTAAATTAGTTGAGGGACTGGACTGTCACACACACACACA
Probe2_EMSA_CRZ1_F	ATTAGAATAGAATATTATCACAGACCCCCCCAATATCTATTTATCAACCT

Probe2_EMSA_CRZ1_R	AGGTTGATAAATAGATATTGGGGGGGTCTGTGATAATATTCTATTCTAAT
Probe3_EMSA_CRZ1_F	TTTCAACTTCCATCATATTTACCACAATTGGATTAACTTGGTTAT
Probe3_EMSA_CRZ1_R	ATAACCAAGTTAATCCAATTGTGGTAAATATGATGGAAGTTGAAA
ChIP_CRZ1_F	GAACCTGTTTGTTTCCTGAGTG
ChIP_CRZ1_R	AGCTAGTATCTTCAATAAATATTTCTGC
